# Methyl 2-amino-3,4,5,6-tetra­fluoro­benzoate

**DOI:** 10.1107/S1600536811015182

**Published:** 2011-05-25

**Authors:** Wei Guo, Xiao-Jian Liao, Guo-Qiang Li, Shi-Hai Xu

**Affiliations:** a Department of Chemistry, Jinan University, Guangzhou 510632, People’s Republic of China

## Abstract

In the title compound, C_8_H_5_F_4_NO_2_, synthesized by esterification of 2,3,4,5-tetra­fluoro­anthranilic acid with methanol, an intra­molecular amine N—H⋯O_carbon­yl_ hydrogen bond is present, while inter­molecular N—H⋯O hydrogen bonds produce chains in the crystal, which extend along the *b-*axis direction.

## Related literature

For general background to this compound and its synthesis, see: Cai *et al.* (1992 ▶); Liao *et al.* (2007 ▶); Xu *et al.* (2008 ▶); Li *et al.* (1999 ▶).
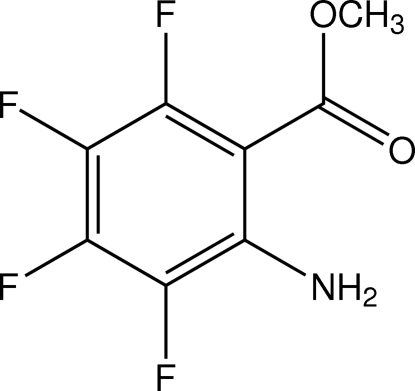


## Experimental

### 

#### Crystal data

C_8_H_5_F_4_NO_2_
*M* *_r_* = 223.0
Monoclinic, 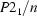

*a* = 4.5246 (2) Å
*b* = 9.6484 (4) Å
*c* = 19.3133 (9) Å
β = 91.324 (4)°
*V* = 842.90 (6) Å^3^
*Z* = 4
Cu *K*α radiation
μ = 1.66 mm^−1^
*T* = 295 K
0.62 × 0.22 × 0.17 mm

#### Data collection

Oxford Diffraction Xcalibur Sapphire3 Gemini Ultra CCD diffractometer
Absorption correction: multi-scan (*CrysAlis PRO*; Oxford Diffraction, 2010 ▶) *T*_min_ = 0.622, *T*_max_ = 1.000
2513 measured reflections
1332 independent reflections
1185 reflections with *I* > 2σ(*I*)
*R*_int_ = 0.018

#### Refinement

*R*[*F* ^2^ > 2σ(*F* ^2^)] = 0.049
*wR*(*F* ^2^) = 0.140
*S* = 1.03
1332 reflections
145 parameters
4 restraints
H atoms treated by a mixture of independent and constrained refinement
Δρ_max_ = 0.17 e Å^−3^
Δρ_min_ = −0.20 e Å^−3^

### 

Data collection: *CrysAlis PRO* (Oxford Diffraction, 2010 ▶); cell refinement: *CrysAlis PRO*; data reduction: *CrysAlis PRO*; program(s) used to solve structure: *SHELXS97* (Sheldrick, 2008 ▶); program(s) used to refine structure: *SHELXL97* (Sheldrick, 2008 ▶); molecular graphics: *OLEX2* (Dolomanov *et al.*, 2009 ▶); software used to prepare material for publication: *OLEX2*.

## Supplementary Material

Crystal structure: contains datablocks I, global. DOI: 10.1107/S1600536811015182/zs2108sup1.cif

Structure factors: contains datablocks I. DOI: 10.1107/S1600536811015182/zs2108Isup2.hkl

Supplementary material file. DOI: 10.1107/S1600536811015182/zs2108Isup3.cml

Additional supplementary materials:  crystallographic information; 3D view; checkCIF report

## Figures and Tables

**Table 1:** Hydrogen-bond geometry (Å, °)

*D*—H⋯*A*	*D*—H	H⋯*A*	*D*⋯*A*	*D*—H⋯*A*
N12—H12*A*⋯O3^i^	0.85 (3)	2.19 (3)	3.028 (2)	171 (3)
N12—H12*B*⋯O3	0.88 (3)	2.00 (3)	2.662 (2)	131 (2)

Symmetry code: (i) 
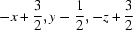
.

**Fig. 1.**
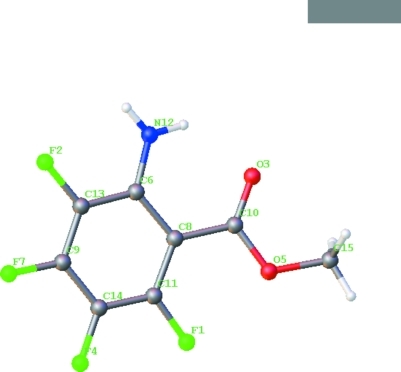
The molecular structure of the title compound showing the atom numbering scheme. Displacement ellipsoids are drawn at the 50% probability level.

## supplementary crystallographic information

### Comment

The title compound C_8_H_5_F_4_NO_2_ (I) (Fig. 1) was prepared by the esterification of 2,3,4,5-tetrafluoroanthranilic acid with methanol (Cai *et al.*, 1992), and is an intermediate product in the synthesis of a coupling reagent (Li *et al.*, 1999; Liao *et al.*, 2007; Xu *et al.*, 2008). In (I), the bond lengths and angles are unexceptional. In the molecule, an intramolecular amine N—H···O_carbonyl_ hydrogen bond is present while intermolecular N—H···O hydrogen bonds give one-dimensional chain structures which extend along the *b* cell direction.

### Experimental

2,3,4,5-Tetrafluoroanthranilic acid (10 mmol) in 50 ml of methanol was cooled in an ice-water bath and 5 ml SOCl_2_ was added dropwise. After 15 min, the mixture was removed and allowed to stand at room temperature for 30 min, and then was refluxed for 8 h. The cooled mixture was washed with 5% Na_2_C03 (1 x 10 ml) and water (2 x 10 ml) and then dried and evaporated to leave 1.71 g (76%) of the title compound as colorless crystals. Crystals suitable for X-ray analysis grew over a period of a week when a solution in methanol was allowed to evaporate in air at room temperature.

### Refinement

The H atoms of the methyl group were positioned geometrically and were included in the refinement in the riding-model approximation, with C—H = 0.96 Å and *U*_iso_(H) = 1.5*U*_eq_(C). The amine H atoms were located in a difference Fourier map and the coordinates and isotropic displacement parameters were refined.

### Crystal data

**Table:**

C_8_H_5_F_4_NO_2_	*F*(000) = 448
*M**_r_* = 223.0	*D*_x_ = 1.758 Mg m^−^^3^
Monoclinic, *P*2_1_/*n*	Cu *K*α radiation, λ = 1.5418 Å
*a* = 4.5246 (2) Å	Cell parameters from 1857 reflections
*b* = 9.6484 (4) Å	θ = 4.6–63.3°
*c* = 19.3133 (9) Å	µ = 1.66 mm^−^^1^
β = 91.324 (4)°	*T* = 295 K
*V* = 842.90 (6) Å^3^	Prism, colourless
*Z* = 4	0.62 × 0.22 × 0.17 mm

### Data collection

**Table:**

Oxford Diffraction Xcalibur Sapphire3 Gemini Ultra CCD diffractometer	1332 independent reflections
Radiation source: Enhance Ultra (Cu) X-ray Source	1185 reflections with *I* > 2σ(*I*)
mirror	*R*_int_ = 0.018
Detector resolution: 16.0288 pixels mm^-1^	θ_max_ = 63.4°, θ_min_ = 4.6°
ω scans	*h* = −5→4
Absorption correction: multi-scan (*CrysAlis PRO*; Oxford Diffraction, 2010)	*k* = −10→10
*T*_min_ = 0.622, *T*_max_ = 1.000	*l* = −22→21
2513 measured reflections	

### Refinement

**Table:**

Refinement on *F*^2^	Primary atom site location: structure-invariant direct methods
Least-squares matrix: full	Secondary atom site location: difference Fourier map
*R*[*F*^2^ > 2σ(*F*^2^)] = 0.049	Hydrogen site location: inferred from neighbouring sites
*wR*(*F*^2^) = 0.140	H atoms treated by a mixture of independent and constrained refinement
*S* = 1.03	*w* = 1/[σ^2^(*F*_o_^2^) + (0.0959*P*)^2^ + 0.1931*P*] where *P* = (*F*_o_^2^ + 2*F*_c_^2^)/3
1332 reflections	(Δ/σ)_max_ = 0.001
145 parameters	Δρ_max_ = 0.17 e Å^−^^3^
4 restraints	Δρ_min_ = −0.20 e Å^−^^3^

### Special details

**Table:**

Geometry. Bond distances, angles etc. have been calculated using the rounded fractional coordinates. All su's are estimated from the variances of the (full) variance-covariance matrix. The cell esds are taken into account in the estimation of distances, angles and torsion angles
Refinement. Refinement of *F*^2^ against ALL reflections. The weighted *R*-factor *wR* and goodness of fit *S* are based on *F*^2^, conventional *R*-factors *R* are based on *F*, with *F* set to zero for negative *F*^2^. The threshold expression of *F*^2^ > σ(*F*^2^) is used only for calculating *R*-factors(gt) *etc*. and is not relevant to the choice of reflections for refinement. *R*-factors based on *F*^2^ are statistically about twice as large as those based on *F*, and *R*- factors based on ALL data will be even larger.

### Fractional atomic coordinates and isotropic or equivalent isotropic displacement parameters (Å^2^)

**Table:**

	*x*	*y*	*z*	*U*_iso_*/*U*_eq_	
F1	−0.0308 (3)	0.34475 (14)	0.54384 (7)	0.0577 (5)	
F2	0.3821 (3)	−0.06985 (13)	0.70892 (7)	0.0608 (5)	
F4	−0.2509 (3)	0.09206 (15)	0.52989 (7)	0.0614 (5)	
F7	−0.0460 (3)	−0.11671 (14)	0.61192 (8)	0.0691 (6)	
O3	0.6161 (4)	0.44489 (16)	0.68851 (8)	0.0594 (6)	
O5	0.3628 (4)	0.50208 (16)	0.59299 (9)	0.0614 (6)	
N12	0.6013 (4)	0.1837 (2)	0.73274 (10)	0.0507 (7)	
C6	0.3985 (4)	0.1679 (2)	0.68073 (10)	0.0394 (6)	
C8	0.3010 (4)	0.2775 (2)	0.63623 (9)	0.0380 (6)	
C9	0.0669 (5)	0.0104 (2)	0.61966 (11)	0.0474 (7)	
C10	0.4398 (4)	0.4146 (2)	0.64304 (10)	0.0408 (6)	
C11	0.0806 (4)	0.2464 (2)	0.58597 (9)	0.0404 (6)	
C13	0.2801 (5)	0.0359 (2)	0.66907 (10)	0.0434 (6)	
C14	−0.0354 (5)	0.1169 (2)	0.57774 (11)	0.0458 (6)	
C15	0.5115 (7)	0.6350 (3)	0.59424 (16)	0.0759 (10)	
H12A	0.661 (7)	0.115 (3)	0.7566 (16)	0.075 (9)*	
H12B	0.678 (6)	0.267 (3)	0.7397 (14)	0.070 (8)*	
H15A	0.44700	0.68880	0.55490	0.1140*	
H15B	0.72120	0.62090	0.59260	0.1140*	
H15C	0.46470	0.68340	0.63600	0.1140*	

### Atomic displacement parameters (Å^2^)

**Table:**

	*U*^11^	*U*^22^	*U*^33^	*U*^12^	*U*^13^	*U*^23^
F1	0.0617 (8)	0.0530 (8)	0.0572 (8)	−0.0006 (6)	−0.0233 (6)	0.0059 (6)
F2	0.0727 (9)	0.0404 (7)	0.0690 (9)	0.0027 (6)	−0.0032 (7)	0.0116 (6)
F4	0.0540 (8)	0.0700 (9)	0.0594 (8)	−0.0116 (6)	−0.0146 (6)	−0.0162 (6)
F7	0.0748 (10)	0.0441 (8)	0.0882 (11)	−0.0209 (6)	−0.0010 (8)	−0.0080 (7)
O3	0.0744 (11)	0.0454 (9)	0.0571 (10)	−0.0116 (7)	−0.0273 (8)	0.0005 (7)
O5	0.0716 (11)	0.0408 (9)	0.0704 (10)	−0.0096 (7)	−0.0279 (8)	0.0127 (7)
N12	0.0606 (12)	0.0402 (11)	0.0505 (11)	0.0041 (9)	−0.0178 (9)	0.0033 (8)
C6	0.0413 (10)	0.0378 (10)	0.0392 (10)	0.0040 (8)	0.0008 (8)	−0.0016 (8)
C8	0.0397 (10)	0.0356 (10)	0.0385 (10)	0.0021 (8)	−0.0039 (8)	−0.0026 (8)
C9	0.0493 (12)	0.0379 (11)	0.0553 (12)	−0.0065 (9)	0.0079 (10)	−0.0071 (9)
C10	0.0433 (11)	0.0354 (10)	0.0433 (11)	0.0041 (8)	−0.0049 (8)	−0.0013 (8)
C11	0.0414 (10)	0.0402 (11)	0.0395 (10)	0.0040 (8)	−0.0039 (8)	−0.0015 (8)
C13	0.0487 (11)	0.0341 (10)	0.0476 (11)	0.0025 (8)	0.0036 (9)	0.0022 (8)
C14	0.0409 (10)	0.0520 (12)	0.0442 (11)	−0.0050 (9)	−0.0038 (8)	−0.0104 (9)
C15	0.095 (2)	0.0390 (13)	0.092 (2)	−0.0168 (13)	−0.0328 (16)	0.0158 (12)

### Geometric parameters (Å, °)

**Table:**

F1—C11	1.341 (2)	C6—C13	1.398 (3)
F2—C13	1.353 (2)	C6—C8	1.426 (3)
F4—C14	1.349 (3)	C8—C11	1.408 (3)
F7—C9	1.336 (2)	C8—C10	1.469 (3)
O3—C10	1.209 (3)	C9—C13	1.364 (3)
O5—C10	1.324 (3)	C9—C14	1.381 (3)
O5—C15	1.448 (3)	C11—C14	1.363 (3)
N12—C6	1.353 (3)	C15—H15A	0.9600
N12—H12A	0.85 (3)	C15—H15B	0.9600
N12—H12B	0.88 (3)	C15—H15C	0.9600
			
C10—O5—C15	115.98 (19)	F1—C11—C8	121.20 (17)
C6—N12—H12B	118.2 (18)	F1—C11—C14	116.08 (17)
H12A—N12—H12B	121 (3)	C8—C11—C14	122.72 (18)
C6—N12—H12A	121 (2)	C6—C13—C9	122.65 (18)
C8—C6—C13	117.80 (17)	F2—C13—C6	118.11 (18)
N12—C6—C8	123.97 (18)	F2—C13—C9	119.25 (17)
N12—C6—C13	118.22 (18)	F4—C14—C9	119.76 (18)
C6—C8—C10	119.22 (16)	F4—C14—C11	120.86 (18)
C6—C8—C11	117.50 (17)	C9—C14—C11	119.4 (2)
C10—C8—C11	123.25 (17)	O5—C15—H15A	109.00
C13—C9—C14	119.87 (19)	O5—C15—H15B	109.00
F7—C9—C13	120.44 (18)	O5—C15—H15C	109.00
F7—C9—C14	119.69 (19)	H15A—C15—H15B	109.00
O3—C10—C8	123.81 (18)	H15A—C15—H15C	109.00
O3—C10—O5	122.31 (18)	H15B—C15—H15C	110.00
O5—C10—C8	113.83 (16)		
			
C15—O5—C10—O3	2.4 (3)	C6—C8—C11—C14	−1.4 (3)
C15—O5—C10—C8	−175.26 (19)	C10—C8—C11—F1	−4.7 (3)
N12—C6—C8—C10	4.4 (3)	C10—C8—C11—C14	176.41 (19)
N12—C6—C8—C11	−177.73 (18)	F7—C9—C13—F2	1.8 (3)
C13—C6—C8—C10	−174.65 (17)	F7—C9—C13—C6	−177.89 (19)
C13—C6—C8—C11	3.3 (3)	C14—C9—C13—F2	−178.81 (19)
N12—C6—C13—F2	−2.2 (3)	C14—C9—C13—C6	1.5 (3)
N12—C6—C13—C9	177.5 (2)	F7—C9—C14—F4	0.8 (3)
C8—C6—C13—F2	176.87 (17)	F7—C9—C14—C11	179.94 (19)
C8—C6—C13—C9	−3.4 (3)	C13—C9—C14—F4	−178.57 (19)
C6—C8—C10—O3	−6.8 (3)	C13—C9—C14—C11	0.6 (3)
C6—C8—C10—O5	170.78 (17)	F1—C11—C14—F4	−0.4 (3)
C11—C8—C10—O3	175.38 (19)	F1—C11—C14—C9	−179.53 (18)
C11—C8—C10—O5	−7.0 (3)	C8—C11—C14—F4	178.59 (18)
C6—C8—C11—F1	177.52 (16)	C8—C11—C14—C9	−0.6 (3)

### Hydrogen-bond geometry (Å, °)

**Table:**

*D*—H···*A*	*D*—H	H···*A*	*D*···*A*	*D*—H···*A*
N12—H12A···O3^i^	0.85 (3)	2.19 (3)	3.028 (2)	171 (3)
N12—H12B···O3	0.88 (3)	2.00 (3)	2.662 (2)	131 (2)
N12—H12A···F2	0.85 (3)	2.36 (3)	2.676 (2)	103 (2)

Symmetry codes: (i) −*x*+3/2, *y*−1/2, −*z*+3/2.
